# ADHD medication in offspring of immigrants — does the income level of the country of parental origin matter?

**DOI:** 10.1186/s12888-017-1572-z

**Published:** 2018-01-08

**Authors:** Arzu Arat, Viveca Östberg, Bo Burström, Anders Hjern

**Affiliations:** 1 Department of Medicine, Karolinska Institute, 171 76, Stockholm, Sweden; 2 Centre for Health Equity Studies (CHESS), Stockholm University/Karolinska Institute, 106 91 Stockholm, Sweden; 3 Department of Public Health Sciences, Karolinska Institute, 171 77 Stockholm, Sweden

**Keywords:** Attention-deficit hyperactivity disorder, Child mental health services, Health inequalities, Immigrant families, Income levels

## Abstract

**Background:**

Child psychiatric treatment facilities vary greatly worldwide and are virtually non-existent in many low-income countries. One of the most common psychiatric disorders in childhood is ADHD, with an estimated prevalence of 3–5% in Sweden. Previous studies have shown a similar prevalence of ADHD in minority and majority children in Sweden and the UK. However, clinical studies demonstrated that children from immigrant families living in Sweden received less psychiatric care than those of native-born parents. We tested the hypothesis that the consumption of child psychiatric care in immigrant families would be determined by the availability of such treatment in the parents’ country of origin. Patterns of medication for attention-deficit hyperactivity disorder (ADHD) were studied as a proxy for child psychiatric care.

**Methods:**

This was a register study of dispensed stimulant medication during 2013–2014 in Swedish national birth cohorts from 1995–2009. The study population, consisting of nearly 1.4 million children, was divided by national income of the parental country of origin and whether the parents were native Swedes, European immigrants, non-European immigrants or a mixture. Logistic regression was used to calculate the odds ratios of having been dispensed at least one ADHD drug during 2013, with adjustments for gender, family status indicating whether the child is living with both parents, household income and area of residence.

**Results:**

Having parents born in low-income (OR [95% confidence interval] 0.27 [0.24–0.29]) or middle-income (European: OR 0.23 [0.20–0.26], non-European: OR 0.39 [0.34–0.41]) countries was associated with lower ADHD treatment levels than having parents born in high-income countries (European: OR 0.60 [0.54–0.66], non-European: OR 0.68 [0.59–0.79]), when compared to children of parents born in Sweden. In families with a background in low or middle income countries, there was no significant association between household income and ADHD medication, while in children with Swedish and mixed backgrounds high level of disposable income was associated with lower levels of ADHD medication.

**Conclusion:**

The use of child psychiatric care by immigrant families in Sweden was largely associated with the income level of the country of origin.

**Electronic supplementary material:**

The online version of this article (10.1186/s12888-017-1572-z) contains supplementary material, which is available to authorized users.

## Background

As with all Nordic welfare societies, Sweden aims to provide equitable access to healthcare for all its citizens, regardless of their gender, socioeconomic situation, region of residence and ethnic, cultural and religious background [1]. The increasing number of immigrants in the country has inspired a number of studies about whether they receive the same equitable access to healthcare as Swedish-born citizens. A recent Danish study found that adult immigrants, their descendants and native Danes did receive equitable access to free healthcare [2], as did a study carried out in Sweden during the 1990s [3].

A clinical study in Sweden however demonstrated differences in the consumption of child psychiatric care between native and immigrant families [4]. Adolescents with immigrant parents from low-income countries were shown to have lower odds of receiving outpatient and inpatient psychiatric care than those with Swedish-born parents. An earlier study led by the same researcher showed that migrant background also played an important role in the referral patterns of adolescents to psychiatric clinics. Children with Swedish parents were more likely to be referred by their own families, whereas children with Asian or African backgrounds were more likely to be referred by social or legal agencies [5].

One of the most common psychiatric disorders in childhood in Western countries is attention-deficit hyperactivity disorder (ADHD), with an estimated prevalence of 3–5% in Swedish school children [6]. Studies in Sweden [7] and Great Britain [8] have shown clear social gradients for ADHD, with a higher prevalence in families with a low socioeconomic status (SES). In contrast, the large national study in Great Britain [8] and a small Swedish population-based study [9] showed that children with a minority background had a very similar prevalence of ADHD to the majority of the population. Interestingly, both a regional Swedish study [10] and national studies in the United States [11] and New Zealand [12] found that minority children were less likely to be diagnosed with ADHD and receive ADHD medication.

Parents play an important role in referring children to psychiatric clinics. Therefore, parental health beliefs, as well as their attitudes, knowledge and experience of mental health services, influence consumption patterns [4, 13, 14]. An analysis of worldwide resources for child and adolescent psychiatry carried out by the World Health Organization (WHO), described huge differences between different global regions [15]. In low-income countries, such resources were often virtually non-existent and they were also scarce in many middle-income countries. This means that immigrant parents from these regions of the world could be expected to have very little experience of such care in their country of origin.

The aim of this study was to test the hypothesis that the developmental level of the parental country of origin, operationalized as low, middle and high-income countries, would be an important determinant of the consumption patterns of child and adolescent psychiatry care by immigrant families in Sweden.

## Methods

This study was based on Swedish national register data held by the National Board of Health and Welfare and Statistics Sweden. A unique identity number with 10 digits is given to all Swedish residents at birth or immigration and this number can be used to link information on those individuals from different register sources.

We tested our hypothesis after controlling for family socio-demographic characteristics. The analysis was further elaborated on by analysing the link between household income and ADHD medication within immigrant background groups.

### Study population

The study population consisted of Swedish born birth cohorts from 1995 to 2009 who were still Swedish residents on 31 December 2012. We excluded individuals who had a diagnosis of narcolepsy in the patient discharge register, because they are prescribed the same medication as children with ADHD. Individuals were also excluded if information on their county of residence, household income, family status or maternal country of birth were missing. A total of 14,405 individuals were excluded, leaving 1,385,397 Swedish born-children in the final study population.

### Immigrant background classification

Table 1 describes the immigrant categories in the study, based on the parental country of birth obtained from the Swedish Register of Total Population. Children having two Swedish parents were recorded as having Swedish background. A mixed category was constructed for children with one Swedish and one foreign-born parent. The rest of the categorization was based on maternal country of birth. The countries were first categorised as European or non-European and then further classified based on gross national income per capita into low, middle and high-income countries [16].

**Table 1:** Nationalities in the immigrant categories in the study

Immigrant Category	Major Nationalities
Sweden (Both parents)	Sweden (100%)
Mixed (One foreign born parent)	European (21,4%), non-European (23,1%)
High income European country	Northern Europe (38%), Eastern Europe (30%), Western Europe (11%), Southern Europe (8%)
Middle income European country	Former Yugoslavia (84%), Romania (7%)
High income non-European country	Chile (49%), Arabian peninsula (13%), North America (11%), South Korea and Japan (8%), Argentina and Uruguay (6%).
Middle income non- European country	Iraq (39%), Turkey (17%), Lebanon (16%), Iran (14%)
Low income non- European country	Somalia (21%), Syria (18%), Ethiopia/Eritrea (17%), South Asia (16%), South East Asia (9%), Northern Africa (7%).

### Outcome variable: ADHD medication

The Swedish Prescribed Drug Register has unique patient identifiers for all drugs prescribed and dispensed to the whole Swedish population since July 2005. The quality of the register has been evaluated and found to be excellent [17]. The first-time purchase of at least one prescription of a drug with an anatomical therapeutic chemical code of N06BA01- N06BA04 between 1 January 2013 and 30 June 2014, was used to create the ADHD medication variable.

### Socio-demographic variables

Socio-demographic variables were created by using the Swedish Longitudinal Integration Database for Health Insurance and Labor Market studies. The unique ID number was used to link information on parental variables from the Swedish Multi-Generation Register. A disposable household income was calculated based on all taxed income and transfers minus taxes paid divided by the number of consumer units in the household. Disposable income of the household is created by Statistics Sweden according to an algorithm that includes all incomes in the household reported to Swedish Tax Agency, subtracted by taxes and divided by consumer units. This variable was then presented in quintiles based on the total sample. Family status was constructed as a dichotomous variable for a child living with a single parent or both parents. Both variables reflected the information when the child was four years old. This was a choice made to prevent inverse causality.

The National Board of Health and Welfare [18] has reported considerable regional differences in the consumption of ADHD medication. Due to the absence of any obvious demographic or geographical patterns in these regional differences, it was assumed that this pattern mirrored varying practices in diagnosis and prescription in different counties rather than variations in the prevalence of ADHD. To account for these differences, the counties were classified into four categories according to the proportion of children prescribed, and whose parents purchased, the ADHD medication during 2013: high prescription (>3.0%), moderate prescription (2.3–3.0%), low prescription (2.1–2.3%) and very low prescription (≤2.0%).

### Statistical analysis

Logistic regression was used to calculate odds ratios (OR) with 95% confidence intervals (CIs) as estimates of effects, with at least one retrieved prescription of ADHD medication as the outcome variable. Two models were used to investigate the association between immigrant background and the incidence of ADHD medication. In Model 1, we entered gender and the child’s age in 2013, broken down into three categories: 4–8, 9–14 and 15–18 years. In Model 2, we added household income, family status and the county of residence, categorised as explained above. In order to estimate the possible effects of child’s gender, an interaction analysis was performed with the immigrant background variable in a logistic regression model that included age and county of residence.

The interaction effects of household income were analysed by calculating odds ratios for ADHD medication in a regression model stratified by household income. Mean length of time spent in Sweden by the parent was calculated for all immigrant backgrounds.

The mean age when ADHD medication was dispensed was calculated to investigate the differences between children with Swedish and immigrant backgrounds. A selection was performed for those who were residents in Sweden on 31 December 2005.

A sensitivity analysis was used to analyse the diagnosis of ADHD in specialist care during 2013. Details on these results are provided in Additional file 1. All statistical analyses were performed using SAS version 9.4 for Windows (SAS Institute Inc., Cary, NC, USA).

## Results

The register entries for the study population, showed that 36,564 children (2.6%) had at least one ADHD drug dispensed to them during the study period, with boys being more likely to receive ADHD medication than girls (3.7% versus 1.7%).

Table 2 presents the study population stratified by immigrant categories. Children from a middle-income European (0.7%) and low income country immigrant background (1.1%) were the least likely to receive medication, compared with children with Swedish (2.8%), mixed (2.9%) or high-income non-European immigrant backgrounds (3.0%).

**Table 2:** Socio-demographic characteristics of the study population: Swedish, Mixed, European (Euro) and Non-European (Non Euro)

	Sweden	Mixed	Euro High	Euro Middle	Non Euro High	Non Euro Mid	Non Euro Low	Total
N	1,058,422	161,924	18,961	30,364	6,996	63,768	44,962	1,385,397
	%	%	%	%	%	%	%	%
Gender								
Boy	51.5	51.3	51.4	51.7	51.4	50.9	51.4	51.4
Girl	48.5	48.7	48.6	48.3	48.6	49.1	48.6	48.6
Age								
15–18	32.0	28.1	28.7	30.1	31.2	26.3	26.4	31.0
9–14	32.8	31.8	26.9	31.0	32.0	30.7	29.6	32.4
4–8	35.2	40.2	44.4	38.9	36.8	43.0	44.0	36.6
Household Income Quintiles								
Lowest	9.7	19.2	29.5	32.5	34.9	54.2	50.7	29.4
2nd	19.2	20.7	21.3	27.5	23.1	22.4	24.0	17.0
3rd	22.0	18.0	15.2	18.6	14.7	9.7	10.9	11.9
4th	23.2	19.0	15.6	13.1	12.8	7.2	7.5	13.3
Highest	25.9	23.2	18.4	8.3	14.6	6.5	7.0	28.4
Single parent								
	11.3	18.8	19.7	15.7	29.5	14.6	26.7	13.2
Years spent in Sweden by parent (mean)								
	–	23.6	19.1	17.2	22.5	17.0	16.7	19.5
ADHD Diagnosis								
	2.1	2.1	1.6	0.5	2.1	1.1	1.0	2.0
ADHD Medication								
	2.8	2.9	2.1	0.7	3.0	1.4	1.1	2.6

It was more common for a child in the Swedish category to belong to one of the two highest income quintiles (49.1%) than immigrant or mixed backgrounds. The percentage declined considerably in children in the non-European middle-income (13.7%) and low-income (14.5%) country categories. Single parenthood was more common in families with immigrant parents and particularly common in those born in high and low-income countries outside Europe (Table 2).

Table 3 presents the incidence of ADHD medication dispensed to boys and girls. Both boys and girls in the European middle-income category had the lowest ADHD medication levels. With regard to children in single parent households, the incidence of ADHD medication was higher for children in the Swedish, mixed or high-income categories.

**Table 3:** Gender stratified socio-demographic indicators and incidence (%) of ADHD medication dispensed by origin

	Swedish	Mixed	Euro High	Euro Middle	Non-Euro High	Non-Euro Mid	Non-Euro Low	Total
BOYS (n)	544,583	83,114	9,758	15,702	3,596	32,453	23,086	712,292
	%	%	%	%	%	%	%	%
Age								
15–18	5.4	6.1	4.8	1.7	5.7	2.7	1.9	5.2
9–14	5.3	5.5	3.9	1.3	5.2	3.1	2.7	5.1
4–8	1.2	1.2	0.9	0.3	1.4	0.7	0.7	1.1
Household Income Quintiles								
Lowest	6.2	5.0	2.6	1.1	3.9	1.9	1.5	4.4
2nd	4.4	4.0	2.5	0.9	3.7	1.9	1.4	4.0
3rd	3.6	3.6	3.0	1.0	2.7	2.1	1.9	3.5
4th	3.4	3.8	2.9	1.1	5.7	2.3	1.5	3.4
Highest	3.4	3.4	3.2	1.0	4.2	2.2	2.0	3.4
Single Parent								
	8.6	7.3	6.1	2.1	5.4	3.3	2.2	7.5
All	3.9	3.9	2.8	1.0	4.0	2.0	1.6	3.7
GIRLS (n)	513,839	78,810	9,203	14,662	3,400	31,315	21,876	673,105
	%	%	%	%	%	%	%	%
Age								
15–18	3.0	3.8	2.8	0.8	3.2	1.3	1.1	2.9
9–14	1.8	1.8	1.5	0.4	2.7	0.9	0.8	1.7
4–8	0.4	0.4	0.2	0.1	0.4	0.2	0.2	0.3
Household income quintiles								
Lowest	2.8	2.0	1.4	0.4	1.4	0.6	0.5	1.9
2nd	1.8	1.7	1.1	0.3	2.2	0.6	0.5	1.6
3rd	1.5	1.7	1.3	0.4	3.3	1.0	0.8	1.5
4th	1.4	1.8	1.9	0.8	1.8	1.3	0.8	1.5
Highest	1.5	1.7	0.6	0.5	2.2	1.0	1.0	1.4
*Single parent*								
	3.9	3.4	2.1	0.8	4.1	1.4	0.9	3.4
All	1.7	1.8	1.3	0.4	2.1	0.7	0.6	1.6

Table 4 shows the results of the multiple logistic regression analyses. Compared to Sweden, children in the European middle-income category had the lowest odds for ADHD medication (OR 0.28), followed by Non-European low-income (OR 0.39) and middle-income countries (OR 0.49). Children in the families with one foreign born parent (OR 1.05) and high-income (OR 0.76) categories had similar odds for ADHD medication as the Swedish category. Adjusting for county of residence and household income (SES) in Model 2 only marginally changed these effects.

**Table 4:** Logistic regression of ADHD medication dispensed

		Model 1	Model 2
	(%)	OR (95% CI)	OR (95% CI)
Sweden	76.4	1	1
Mixed	11.7	1.05 (1.02–1.08)	0.90 (0.88–0.93)
European high	1.4	0.76 (0.68–0.84)	0.60 (0.54–0.66)
European middle	2.2	0.28 (0.25–0.32)	0.23 (0.20–0.26)
Non-European high	0.5	1.00 (0.86–1.14)	0.68 (0.59–0.79)
Non- European middle	4.6	0.49 (0.46–0.53)	0.39 (0.36–0.41)
Non- European low	3.3	0.39 (0.36–0.43)	0.27 (0.24–0.29)

Model 1 is adjusted for age and gender

Model 2 is adjusted for age, gender, income, county of residence and lone parenthood

Table 5 presents the odds ratios for ADHD medication dispensed by income quintiles in the parental country of birth categories. Swedish children and children with a mixed background who lived in a household with higher income were less likely to receive ADHD medication compared to children with same background living in households with lower income. This stepwise social gradient was absent in all other immigrant background groups.

**Table 5:** Logistic regression for ADHD medication by household income level within each immigrant category

	Lowest Income	2nd Income Quintile	3rd Income Quintile	4th Income Quintile	Highest Income
		OR (95% CI)	OR (95% CI)	OR (95% CI)	OR (95% CI)
Sweden	1	0.88 (0.85–0.91)	0.74 (0.72–0.78)	0.65 (0.62–0.67)	0.51 (0.49–0.53)
Mixed	1	0.97 (0.88–1.06)	0.85 (0.78–0.94)	0.81 (0.74–0.89)	0.61 (0.56–0.67)
European high	1	0.99 (0.73–1.33)	1.07 (0.78–1.48)	1.06 (0.78–1.44)	0.78 (0.58–1.07)
European middle	1	0.98 (0.68–1.41)	1.12 (0.76–1.65)	1.23 (0.83–1.84)	0.73 (0.43–1.22)
Non-European high	1	1.16 (0.79–1.70)	1.06 (0.68–1.65)	1.18 (0.77–1.83)	0.89 (0.58–1.38)
Non- European middle	1	1.13 (0.95–1.35)	1.20 (0.96–1.50)	1.17 (0.92–1.50)	0.91 (0.70–1.19)
Non- European low	1	0.97 (0.77–1.23)	1.21 (0.92–1.60)	0.87 (0.62–1.23)	0.96 (0.70–1.34)

Model is adjusted for age, gender, county of residence and lone parenthood

In a sensitivity analyses that had an ADHD diagnosis in specialised care as the outcome, the multiple logistic regression gave similar results as the medication outcome (Additional file 1: Table S1). Compared to children with native parents, children in the European middle-income category had the lowest odds of receiving an ADHD diagnosis (OR [95%CI]: 0.32 [0.28–0.37]), followed by the non-European low (0.52 [0.47–0.57]) and middle- income countries (0.59 [0.55–0.64]). Children of families with one parent foreign country background and European and non-European high-income categories had similar odds for ADHD diagnosis as the Swedish category with ORs of 1.10 [1.0–1.14] and 0.87 [0.78–0.98] respectively. When adjusted for county of residence and disposable income (SES) in Model 2, the odds were only marginally changed. The lower figures for diagnosis compared to medication levels was due to the prescriptions by practitioners who do not report to the Swedish Patient Register.

The mean age for first ADHD diagnosis and first medication was calculated for all immigrant backgrounds and for both genders and no major differences were observed between the populations (data not shown). The mean of length of stay of parent was calculated for all migrant groups. Since length of stay of the mother was strongly nested with the child’s age, there was large variation in the number of years spent in Sweden (data not shown). Mean length of stay was 19.5 years and figures were only slightly different across groups (Table 2).

## Discussion

This register-based study of nearly 1.4 million Swedish children demonstrates that children whose parents were born in countries where there were few child psychiatric resources, namely the non-European low and middle-income countries, were less likely to receive ADHD medication than children with parents born in Sweden or other high-income countries. For the children of Swedish-born and mixed parents, ADHD medication levels were higher for children in households with low level of disposable income, where as in families with an origin in low or middle income countries, there was no such gradient between household income and ADHD medication.

The observed pattern of ADHD medication and diagnosis by immigrant categories is likely to reflect the facilities available for child and adolescent psychiatry in the parents’ country of origin. According to a report by the WHO [15] child and adolescent mental health care is scarce in countries that are in the lowest World Bank income categories. It seems very probable that the presence, or absence, of child psychiatry in the country of origin influences parental belief systems around mental health and behaviour in children and consequently their attitudes towards seeking help. The influences of parental country of origin on attitudes towards behavioural disorders have been covered by several previous studies. One study found that immigrant parents from Morocco, Turkey and Suriname who were living in The Netherlands had lower sensitivity, but higher specificity, for ADHD detection rates, than Dutch parents [14]. This suggests that a higher degree of deviance was present before ADHD was considered by these immigrant parents. Another study found that Pakistani women living in the UK were less likely to access mental health services for conditions judged as mild or moderate [19].

Compared to Swedish-born and mixed families, we expected a reversed social gradient in families in the low-income and middle-income countries with respect to household income and ADHD medication levels. This would fit a cross-cultural pattern, where a higher education, and consequently higher income, often leads to more familiarity with the belief systems associated with Western child psychiatry [20]. Also, higher income indicates more stable integration into the job market, leading to higher knowledge of the Swedish healthcare system and of ways to seek help for children’s health problems. Absence of this social gradient in our study population underlines the importance of availability of child psychiatry services in the country of origin for use of child psychiatry services in Sweden.

We cannot exclude the possibility of a healthy migrant effect behind the lower rates of ADHD diagnosis and medication consumption in children with an immigrant background. Family, twin and adoption studies provide robust evidence for strong genetic influences on ADHD [21, 22], which makes a low prevalence of parental ADHD a possible mechanism for a healthy migrant effect that carries over to the next generation. However, the clear patterns observed related to gross national income per capita in the parental country of birth and parental socioeconomic status in Sweden cannot easily be explained by a healthy migrant effect. Therefore, this points to a more structural explanation.

### Strengths and limitations

To the best of our knowledge, this is the first study that has tested the hypothesis that the availability of child psychiatric services in the country of origin was an important determinant of consumption of such care after migration. This was made possible by our large national cohort, where foreign-born parents could be divided into categories based on the developmental level of their country of origin and these categories could be further divided by their income level in Sweden.

The study has several limitations. According to national guidelines for ADHD medication, specialists are only advised to prescribe stimulants in cases where other interventions have failed. Therefore, only the more severe cases of ADHD are captured by medication levels. However, a sensitivity analysis of children diagnosed in specialised care confirmed the pattern found for medication in our study, making it improbable that this bias would have had a major impact on our study.

At the time of the study medication was not free for children, as it is today. This might be expected to explain the differences in levels of medication dispense between lower and higher household income, for the children with migrant low and middle income country background. However, sensitivity analysis with diagnosis levels shows this to be less likely since there were no costs for medical visits for children.

Teachers play an important role in referring children with behavioural problems, like ADHD, to child psychiatrists, since the symptoms of ADHD often interfere with school work. Labelling child behavioural problems in the school setting can easily be influenced by preconceptions about the children’s ethnic background, leading to biased referral patterns. Our study showed a clear gradient of consumption of care by the gross national income per capita of the parental country of origin. This gradient also existed between children in families with a non-European heritage, such that children in the non-European high income category had higher medication levels compared to low and middle income country backgrounds. Thus, preconceptions about children with a non-European background seem unlikely to explain this pattern.

## Conclusions

The observed pattern of ADHD medication and diagnosis by immigrant categories might reflect the facilities available for child and adolescent psychiatry in the parents’ country of origin, and thus their familiarity with child psychiatric services. Child psychiatric services need to find ways of reaching out to immigrant families with this background in order to provide equitable child psychiatric care.

## Additional file

Additional file 1: Table S1. Logistic regression of ADHD diagnosis (DOCX 12 kb)

## Abbreviations

ADHD: Attention-deficit hyperactivity disorder
CI: Confidence interval
OR: Odds ratio
SES: Socioeconomic status
WHO: World Health Organization
