# Supplementary material for: ADHD medication in offspring of immigrants — does the income level of the country of parental origin matter?
Source: BMC Psychiatry. 2018 Jan 8;18:3. doi: 10.1186/s12888-017-1572-z (PMC5759312; doi:10.1186/s12888-017-1572-z)
Supplement: Additional file 1: Table S1. — Logistic regression of ADHD diagnosis (DOCX 12 kb) [file 12888_2017_1572_MOESM1_ESM.docx]

| Table S1. Logistic regression of ADHD diagnosis. | | | |
| --- | --- | --- | --- |
|  |  | Model 1 | Model 2 |
|  | (%) | OR (95% CI) | OR (95% CI) |
| Sweden | 76.4 | 1 | 1 |
| Mixed | 11.7 | 1.10 (1.0-1.14) | 0.95 (0.92-0.99) |
| European high | 1.4 | 0.87 (0.78-0.98) | 0.70 (0.63-0.79) |
| European middle | 2.2 | 0.32 (0.28-0.37) | 0.27 (0.23-0.30) |
| Non-European high | 0.5 | 1.04 (0.88-1.22) | 0.73 (0.62-0.86) |
| Non-European middle | 4.6 | 0.59 (0.55-0.64) | 0.47 (0.44-0.51) |
| Non-European low | 3.3 | 0.52 (0.47-0.57) | 0.36 (0.33-0.40) |

**Additional file 1**

Model 1 is adjusted for age and gender

Model 2 is adjusted for age, gender, income, county of residence and lone parenthood.
